# Tackling Anxiety‐ and Stress‐Related Freezing of Gait in People With Parkinson's Disease (TACKLING‐FOG): Study Protocol for a Randomized Controlled Trial

**DOI:** 10.1111/ejn.70584

**Published:** 2026-06-17

**Authors:** Gijs Vissers, Anouk Tosserams, Annelien A. Duits, Anouk van der Heide, Bastiaan R. Bloem, Rick C. Helmich, William R. Young, Jorik Nonnekes

**Affiliations:** ^1^ Donders Institute for Brain Cognition and Behavior; Department of Rehabilitation; Center of Expertise for Parkinson & Movement Disorders Radboud University Medical Center Nijmegen the Netherlands; ^2^ Donders Institute for Brain Cognition and Behavior; Department of Neurology; Center of Expertise for Parkinson & Movement Disorders Radboud University Medical Center Nijmegen the Netherlands; ^3^ Department of Medical Psychology Radboud University Medical Centre Nijmegen the Netherlands; ^4^ Department of Psychiatry and Neuropsychology Maastricht University Maastricht the Netherlands; ^5^ Donders Institute for Brain Cognition and Behavior; Centre for Cognitive Neuroimaging Radboud University Nijmegen the Netherlands; ^6^ School of Sport and Health Sciences University of Exeter Exeter UK

**Keywords:** anxiety, design, freezing of gait, Parkinson's disease, protocol, randomized controlled trial, rehabilitation

## Abstract

Freezing of gait (FOG) is a common and disabling symptom in people with Parkinson's Disease (PD), characterized by paroxysmal episodes where there is an inability to step effectively, despite attempting to do so. Anxiety and stress exacerbate FOG, particularly in situations where people with FOG anticipate not being in control of their movements. Some people with PD use compensatory strategies that target anxiety and stress to improve FOG. However, tailored strategies to ameliorate anxiety‐ and stress‐related FOG have never been evaluated in a systematic manner. We describe the study protocol of the TACKLING‐FOG trial, which aims to evaluate the effectiveness of a novel 4‐week Managing the Mental State intervention in reducing FOG‐related anxiety and stress to improve FOG. We also aim to identify the key determinants influencing the response to the intervention. This study is a randomized controlled trial (RCT) with a waitlist control group. Forty participants with PD who experience anxiety‐ or stress‐related FOG will be included. All participants receive a baseline measurement and are subsequently randomized to the intervention or control group in a 1:1 ratio. The intervention group immediately receives four weekly sessions of the Managing the Mental State intervention, whereas the control group enters a waitlist period. Afterwards, both groups are reassessed. Next, the intervention group enters a 10‐week follow‐up period, while the control group receives the intervention, is reassessed, and enters the follow‐up period. Both groups receive a final measurement at the end of the follow‐up. The primary outcome is the percentage of time frozen during a home‐based walking course that includes self‐selected FOG “hotspots.” Secondary outcomes involve the percentage of time frozen during the same trajectory under elevated stress conditions and during a standardized FOG‐provoking protocol. Additionally, heart rate is collected as a physiological marker of stress and anxiety, and questionnaires are administered to assess domains that may improve in response to the intervention, including anxiety, quality of life, and self‐esteem. TACKLING‐FOG will be the first RCT to examine the use of tailor‐made behavioral strategies to tackle anxiety‐ and stress‐related FOG in people with PD.

**Trial Registration:** Clinicaltrials.gov NCT06302309. Registered on March 8, 2024

## Graphical abstract

Participants undergo baseline assessment followed by randomization to either the intervention or a waitlist control condition. The intervention group receives four sessions immediately, while the waitlist group enters a 4‐week waiting period. Post‐intervention assessment is conducted in both groups, after which the waitlist group crosses over to receive the intervention. A final follow‐up assessment takes place 10 weeks after intervention completion in both groups.

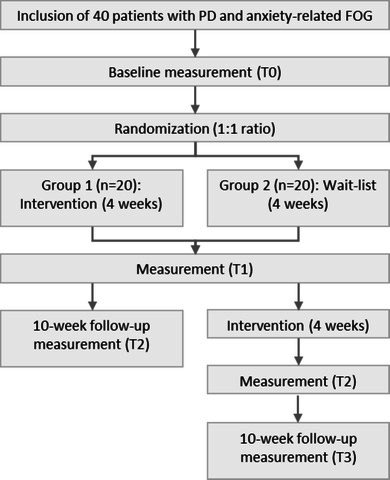

**Abbreviations**
CISS‐21: Coping Inventory for Stressful Situations‐21
DBS: deep brain stimulation
FOG: freezing of gait
MoCa: Montreal Cognitive Assessment
NFOG‐Q: New Freezing of Gait Questionnaire
NRS: Numeric Rating Scale
PD: Parkinson's disease
PDQ‐39: Parkinson's Disease Questionnaire‐39
RCT: randomized controlled trial
UPDRS: Unified Parkinson's Disease Rating Scale

## 1 Introduction

### 1.1 Background

Freezing of gait (FOG) is one of the most debilitating symptoms of Parkinson's disease (PD). FOG is clinically characterized by paroxysmal episodes wherein there is an inability to step effectively, despite attempting to do so (Gilat et al. 2026; Weiss et al. 2020). It is estimated that FOG impacts the lives of roughly half of all people with PD (Zhang et al. 2021). FOG impairs quality of life in patients and is an important risk factor for falls (Canning et al. 2014; Walton et al. 2015). It is often difficult to treat FOG satisfactorily with pharmacological intervention, highlighting the importance of rehabilitation and approaches for self‐management (Tosserams et al. 2025; Nonnekes et al. 2015). However, current approaches including cueing and movement strategies only partially alleviate FOG.

In addition to common triggers such as narrow spaces and turning motions, anxiety and stress are increasingly being recognized as important triggers for FOG (Conde et al. 2023). In fact, evidence of a specific FOG subgroup of so‐called “anxious freezers” is accumulating. For example, an observational study was able to subtype participants based on their vulnerability to specific triggers, identifying a subgroup of “anxious freezers” who mainly experience FOG in stressful or hurried situations. In contrast, “asymmetric‐motor” freezers experience FOG mostly while turning and passing through doorways, and “sensory‐attention” freezers experience FOG in situations that require shifting attention (Martens et al. 2018). Furthermore, physiological markers of anxiety and stress, such as heart rate and galvanic skin response, increase just before the occurrence of a freezing episode, supporting the contributing role of anxiety and stress in the occurrence of FOG (Economou et al. 2021; Mazilu et al. 2015). Also, experimental studies have demonstrated that anxiety and stress can trigger FOG. For example, during a virtual reality paradigm, participants spent significantly more time frozen when maneuvring a plank that appeared to be high above a pit, compared to walking over a plank that appeared to be on floor level (Martens et al. 2014). While the neural mechanisms underlying the relationship between anxiety and stress with FOG remain to be fully elucidated, it has been hypothesized that the increase in physical arousal that accompanies anxiety and stress might “set the stage for freezing” by promoting dysfunctional cross‐talk between multiple cortical networks, which ultimately results in FOG (Taylor et al. 2022).

The relationship between anxiety and stress with FOG manifests across a range of contexts. This includes anxieties or stressors relating to (i) time pressure (e.g., trying to reach to a ringing phone before the caller hangs up), (ii) social/presentational concerns (e.g., concern about judgment of onlookers as one tries to board public transport), and (iii) fear of falling. Each of these examples shares a common theme: that people with FOG often anticipate situations where they will not be in control of their movements. Although people with PD are often well aware that the feelings and situations mentioned above can significantly worsen or provoke their FOG, they are rarely aware of behavioral strategies that could improve their mental state and potentially reduce FOG, and therefore do not actively use them. In a survey study including 4324 people with PD, among those who used compensatory strategies in daily life, about 70% of respondents reported using compensatory strategies that target anxiety and stress to reduce FOG (Tosserams et al. 2021). Examples of these strategies include using positive affirmations, practicing breathing exercises, and avoiding feeling rushed by others (Tosserams et al. 2023). Interestingly, the effectiveness of each of these strategies varied markedly per person and depended on the context in which they were applied. These findings put forward the potential of adopting a personalized approach to reduce anxiety‐ and stress‐related FOG. As such, the treatment of anxiety‐ and stress‐related FOG could benefit from an intervention in which people with PD are guided in selecting personalized strategies for managing FOG and associated anxiety and stress, based on their specific needs and circumstances.

These considerations inspired the development of a behavioral intervention, in which the previously mentioned compensatory strategies are offered in a structured and personalized manner. During this “Managing the Mental State” intervention, participants will first receive psychoeducation on how anxiety, stress, and worrisome thoughts exacerbate FOG. Next, the contexts that trigger anxiety‐ and stress‐related FOG are explored and identified. This is used as a basis to select personalized strategies aimed at reducing FOG‐related anxiety and stress. The effectiveness of the Managing the Mental State intervention will be evaluated in a randomized controlled trial, the protocol of which is presented here. By publishing this study protocol, we aim to ensure methodological transparency and provide a reproducible framework for the trial, which will be cited in the forthcoming results publication.

### 1.2 Objectives

This study aims to evaluate whether a nonpharmacological, tailored intervention targeting FOG‐related anxiety and stress is effective in reducing FOG in people with PD. Specifically, we aim to study:

1. the effect of four sessions of a Managing the Mental State intervention in people with disabling and anxiety‐ and stress‐related FOG;
2. the key determinants of the effectiveness of the intervention.

### 1.3 Hypothesis

We predict that the percentage of time that participants spent frozen while walking a personalized walking course that includes self‐selected FOG “hotspots” will be significantly reduced after the intervention compared to usual care, with retained effects at a 10‐week follow‐up.

## 2 Methods

### 2.1 Regulation Statement

TACKLING‐FOG will be conducted according to the principles of the Declaration of Helsinki (64th WMA General Assembly, Fortaleza, Brazil, October 2013) and the Medical Research Involving Human Subjects Act. The protocol is written in accordance with the SPIRIT 2013 checklist. The study has received ethical approval from the accredited medical ethical committee METC Oost‐Nederland, the Netherlands (NL85217.091.23). Any protocol modifications will be notified to the committee via amendments. The trial has also been registered on Clinicaltrials.gov (NCT06302309). All participants will sign informed consent prior to data collection by the assessor (MH) on the day of the first measurements.

### 2.2 Study Design and Setting

TACKLING‐FOG is a 4‐week, monocenter, two‐armed, randomized controlled trial with a waitlist control group. The study will be conducted by the Department of Rehabilitation within the Centre of Expertise for Parkinson's Disease of the Radboud University Medical Center (Radboudumc), Nijmegen, The Netherlands. The measurements and the first two sessions of the intervention will take place in the home setting of the participants; the last two sessions will be held remotely. However, if conducting the sessions remotely is unfeasible (e.g., if the participants are not able to), they will be held at the participants' homes. The structure of the intervention sessions is consistent, whether held online or home‐based, so this is not formally documented, nor included in the analysis.

### 2.3 Recruitment and Selection

Forty participants will be recruited via the Parkinson recruiting platform ParkinsonNEXT (https://www.parkinsonnext.nl/), as well as via the research page of the Dutch patient organization for people with PD (https://www.parkinson‐vereniging.nl/). In addition, participants will also be recruited via the large outpatient clinic of the Centre of Expertise for Parkinson's disease, and from an existing cohort of people with PD that previously participated in studies conducted at the Department of Rehabilitation, Radboudumc. Here, eligible subjects are contacted if they formally indicated on the relevant consent form a willingness to be contacted regarding future studies. People with PD who have expressed their interest (by contacting the researcher via email or phone) will receive an information letter from the assessor of the study. After a week, the assessor will contact the people who expressed their interest and confirm if they would still like to participate. If the patients agree to participate, eligibility is checked by going through a list of predefined questions. After inclusion, participants may withdraw from participating in the study at any given time.

### 2.4 Eligibility

In order to be eligible to participate in this study, a participant must meet all of the following criteria:

- Idiopathic PD, as diagnosed according to the MDS criteria (Postuma et al. 2015);
- Occurrence of FOG multiple times a day (as objectified with the new freezing of gait questionnaire) (Nieuwboer et al. 2009), that is related to anxiety or stress (positive answer to the question: Does FOG occur or get worse when you are anxious or stressed?);
- Using a stable dose of PD medication and/or stable deep brain stimulation (DBS) settings for the duration of the trial. However, adjustments of PD medication and DBS settings during the trial are allowed if deemed clinically necessary.

A potential participant who meets any of the following criteria will be excluded from participating in the study:

- Any comorbidity (i.e., neurological and orthopedic) that significantly impacts gait;
- Severe cognitive impairment hampering the provision of informed consent, or the ability to comply with the study protocol, including understanding instructions during intervention and study assessments. No formal cognitive threshold was applied, as the study was designed to be broadly inclusive.

### 2.5 Group Allocation and Blinding

Participants will be allocated to the intervention group or to the waitlist control group in a 1:1 ratio, based on stratified block randomization using variable block sizes (*n* = 4–6). Stratification will be based on the percentage of time a participant is frozen during a personalized walking course designed to elicit FOG (described in detail in Section 2.10), prior to randomization, with a cut‐off of 15%. This cut‐off was selected based on clinical experience to distinguish between relatively mild and more pronounced FOG severity. Randomization will be performed by the assessor in CastorEDC, a web‐based data management system for academic studies (http://www.castoredc.com). Participants cannot be blinded as they are aware of whether they receive the intervention or enter the waitlist period. The assessor cannot be blinded either, as during the second measurement participants are instructed to apply the strategies taught during the intervention (e.g., breathing exercise or listening to one's favorite music), making it apparent whether a strategy is being used. However, to minimize bias, measurements are conducted by an independent assessor, whereas the intervention is provided by a neuropsychologist.

### 2.6 Participant Timeline

**FIGURE 1:**
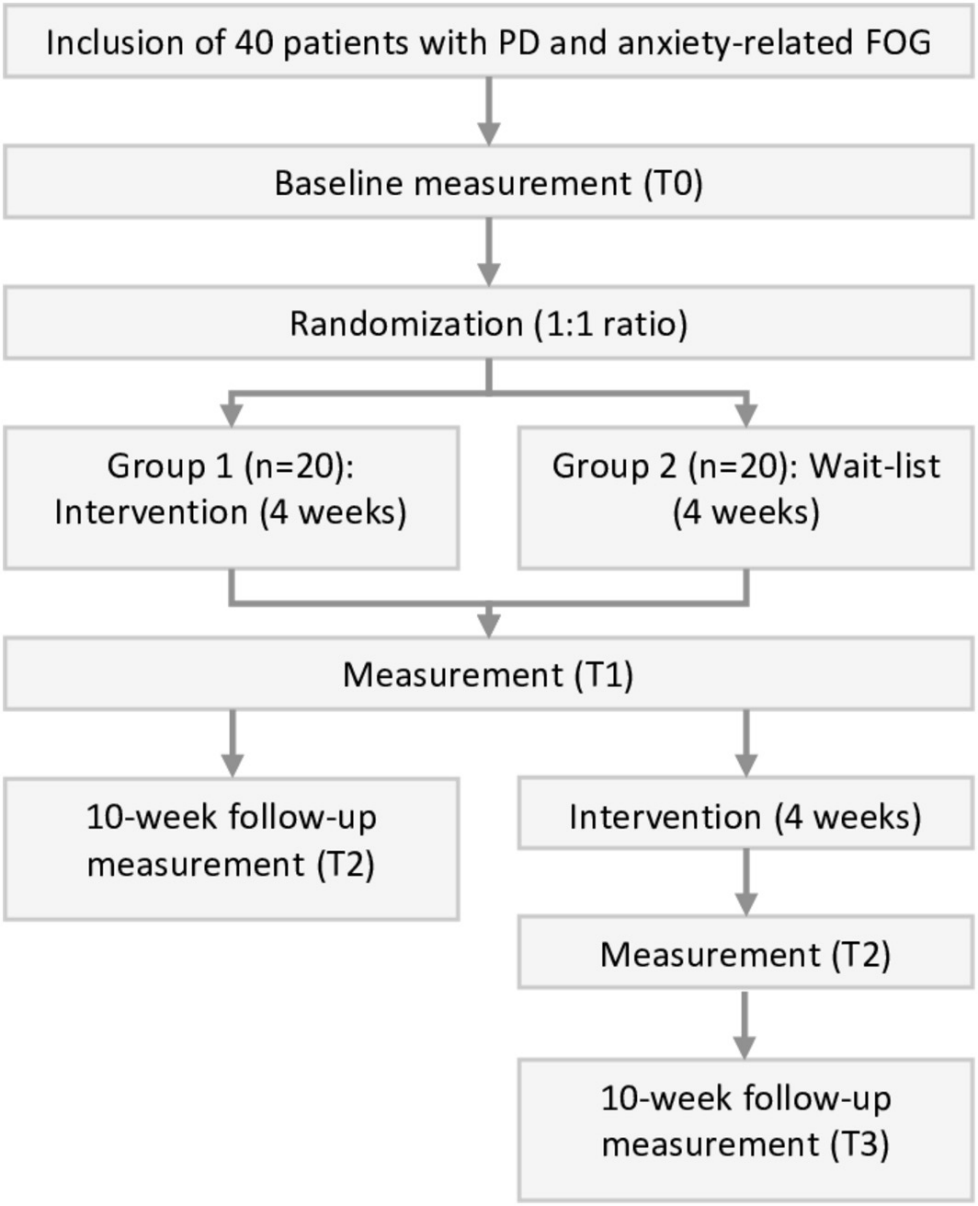
Study flowchart.

The timeline of this study is shown in Figure 1. Following inclusion, participants will receive a baseline measurement. After the baseline measurement, participants will be allocated randomly to the intervention group or to the waitlist control group. Immediately after randomization, the intervention group will receive four sessions of the Managing the Mental State intervention, whereas the waitlist control group will enter a 4‐week waiting period. Participants will receive the intervention or enter the waitlist individually as soon as they are randomized. After the intervention or waitlist period, both groups will receive a second measurement. After this second measurement, the intervention group will enter the follow‐up period, while the waitlist control group starts the intervention. Immediately after completing the intervention, the waitlist control group receives a third measurement and enters the follow‐up period. Both groups will receive a final measurement 10 weeks after completion of the intervention. The number of 10 weeks for the follow‐up period was chosen because clinically meaningful progression in PD is generally limited within this timeframe, thereby reducing the risk of confounding. In addition, this duration was considered feasible for participants and represents a pragmatic choice to obtain an initial indication of the retention of treatment effects after the intervention.

### 2.7 Intervention: Managing the Mental State Intervention

The Managing the Mental State intervention is a psychological treatment that aims to reduce FOG‐related anxiety and stress, as well as their impact on FOG by (1) providing psychoeducation on the impact that anxiety and stress can have on FOG, (2) identifying the contexts in which participants perceive anxiety and stress related to FOG, and (3) teaching participants tailored strategies to reduce the impact that anxiety and stress have on FOG (see Table 1). The intervention consists of four sessions. All participants will follow the same structured session format; however, the duration of each session will vary between 60 and 90 min depending on the individual participant and the specific strategies being practiced. The exact session length will not be documented. The first two sessions take place in the home setting of the participants. The remaining sessions are preferably conducted remotely through a videocall. However, in the case that this is not possible due to the technological requirements that are involved, the last sessions also take place in the home setting. If possible, the partners or caregivers of the participants are also involved in the treatment, as they can provide support and encouragement to the participants, thereby promoting a successful outcome of the intervention.

**TABLE 1:** General outline of the sessions.

Session	Parts of the session
**Session 1**	Psychoeducation: Verbal explanation and patient video Identify FOG contexts Home assignment: Diary keeping
**Session 2**	Reviewing diary Education on behavioral strategies Selecting and practicing strategies Home assignment: Practicing selected strategies
**Session 3**	Evaluation of strategies Selecting which strategies to retain and replace Practicing new strategies
**Session 4**	Evaluation of strategies Selecting final strategies Discussing whether and how to maintain strategy use Evaluation of intervention

The intervention will be conducted by the first author (GV) who is an experienced neuropsychologist. He will deliver the intervention to all participants to ensure consistency across participants. No other individuals will be involved in delivering the intervention. The Managing the Mental State protocol has been developed in conjunction with researchers and clinicians specialized in the treatment of gait impairments, as well as experts on the psychological treatment of anxiety in PD. The psychoeducation material has been developed in collaboration with a “project advisory group” made up of people with PD and FOG, their family members, and specialist physicians/clinicians, and was specifically designed for the present study. The intervention was tested in a small pilot in three people with PD, which confirmed that this number of sessions was feasible.

#### 2.7.1 Session 1: Psychoeducation and Identifying Individual Triggers

The first session will start by providing psychoeducation. For this purpose, the therapist first verbally explains what anxiety and stress are, and how they can impact FOG. Subsequently, participants will watch a video in which people with PD share their experience about anxiety‐ and stress‐related FOG, with the aim of creating recognition and supporting a process where participants recognize similar thoughts and feelings in others, which in turn creates a sense of shared understanding. Subsequently, the contexts in which anxiety‐ and stress‐related FOG occurs will be mapped by asking participants to reflect on situations that elicit FOG‐related anxiety and stress. To identify worrisome thoughts associated with freezing that may be related to anxiety, participants are asked to walk in locations within their home environment where freezing commonly occurs, and to immediately recall their thoughts (Young et al. 2020). Lastly, participants are asked to keep a diary as a therapeutic tool to increase awareness of situations and worrisome thoughts that accompany anxiety‐ and stress‐related FOG and to increase their ability to recognize these. Specifically, they will be asked to keep track of the situations in which FOG occurred throughout the week, as well as the thoughts and feelings that preceded these episodes.

#### 2.7.2 Session 2: Introducing Managing the Mental State Strategies

During the second session, participants are educated about the Managing the Mental State compensation strategies. These strategies are based on a previous study, which documented various ways in which people with PD try to overcome their gait impairments by targeting anxiety and stress (Tosserams et al. 2021, 2023). For the purpose of the current study, the initial list has been revised in conjunction with expert (neuro)psychologists. The final list includes strategies chosen for their practical applicability and integration within cognitive behavioral therapy (see Table 2). Some aspects of the strategies are adapted from a cognitive behavioral treatment protocol for anxiety in PD by Moonen et al. (2021). The therapist determines which strategies to use based on their clinical experience, taking into account factors such as the type of trigger (e.g., time pressure or fear of falling), the possible role of excessive worrying thoughts, and the participant's preferred type of strategy. The therapist will start by providing an overview of the complete list of available strategies. Based on the contexts in which the participants experience anxiety or stress‐related FOG, the therapist suggests strategies to try out that fit the situation. For example, if a patient experiences physical tension prior to a freezing episode, performing a relaxation exercise can be helpful. Alternatively, if a participant experiences social pressure, a strategy involving communicating beforehand how one is feeling might be preferable. Since the effectiveness of compensation strategies varies considerably from person to person, and it is not yet possible to determine which strategy is effective for a specific individual, multiple strategies are practiced (Tosserams et al. 2021). The therapist and participants collaboratively select two initial strategies to practice, taking into account any strategies that are preferred by the participants. Subsequently, the therapist will first provide a demonstration on how to execute the selected strategies, which is followed by joint practice during the session. If either the participant or the therapist perceives a strategy to be ineffective, an alternative strategy is chosen. Afterwards, the participants are asked to practice the strategies at home in‐between sessions. Participants are encouraged to practice these strategies every day until the next session. They will also receive a diary to document their strategy use, including when and for how long they practiced each strategy and whether it was effective. This diary will be used during the later sessions of the intervention to review and discuss their home practice over the week and serves solely as a therapeutic tool rather than an outcome measure.

**TABLE 2:** List of managing the mental state strategies (Tosserams et al. 2023).

Categories	Behavioral strategies	Explanation
Facilitating general relaxation	Deep breathing exercise	During the deep breathing exercise, participants learn to control their anxiety response by changing their way of breathing. Specifically, participants are instructed to slow down breathing and change the way in which they breathe, namely, by taking deeper diaphragmatic breaths through their nose (Hopper et al. 2019).
	Shift attention	People who experience anxiety often tend to focus on their anxious thoughts and feelings. Therefore, redirecting attention outward can have a calming effect and may enhance participants' perceived level of control over the situation. During this exercise, participants are instructed to divert their attention away from their body and instead pay attention to the sights, sounds, and scents around them (Moonen et al. 2021).
	Body scan	The body scan is an accessible relaxation technique that aims to foster relaxation and reduce stress and anxiety. During this exercise, participants are instructed to bring their attention towards their body, as they “scan” each part of their body one by one, from head to toe (Call et al. 2014).
	Muscle relaxation exercise	Progressive muscle relaxation is a relaxation exercise during which tension is produced in specific muscle groups, which is followed by relaxation of the muscles. During the exercise, participants are asked to tighten each muscle group one at a time, starting at the lower extremities and working towards the head and neck (Donato et al. 2026).
	Listening to one's favorite music	Participants may listen to their favorite music during situations in which they experience feelings of anxiety or stress that surrounds freezing of gait. Participants may choose music that is either calm or somewhat more upbeat, which they enjoy listening to.
Eliminating negative emotions and cognitions surrounding FOG	Coping statements	A coping statement is a statement that can be used when confronting situations in which worrisome thoughts about FOG are present that may elicit anxiety or stress‐related FOG (e.g., worrying about falling or thinking about the judgment of onlookers while walking). This strategy encourages participants to reframe worrisome thoughts that are present in the relevant situation. The thought is reframed into a more helpful and balanced thought in the form of a concrete statement that the participant can use when dealing with the anxiety‐ or stress‐provoking event. The goal of the exercise is not simply to think positively, but to formulate a more realistic and helpful thought (Moonen et al. 2021).
	Focus on what you can control	During this strategy, participants are encouraged to explicitly try to focus on the factors they have control over during situations that provoke freezing, instead of focusing on things they cannot control. Things they have control over might include focusing on the task at hand and their behavior. What they cannot control may include their surroundings or the behavior of others.
	Thought stopping	During thought stopping, participants are encouraged to actively try to stop engaging in worrisome thoughts that are present in situations that elicit FOG. During the anxious or stressful situation, participants are instructed to actively try to stop FOG‐related worrisome thoughts by saying “stop,” imagining a big red stop sign and subsequently focusing on their surroundings (Moonen et al. 2021).
	Positive imagery	Participants are asked to recall or envision a scenario that evokes feelings of happiness or tranquility. This image is then utilized during the stressful or anxious situations to foster these positive feelings to try reducing feelings of anxiety or stress that are present right before or during a freezing episode (Kumari and Patil 2023).
	Visualizing a successful outcome	Participants are encouraged to visualize a situation that triggers freezing of gait. They are then asked to imagine walking toward the place that elicits FOG and visualizing their movements as successful. They are encouraged to visualize the scene as vividly as possible, engaging their senses and emotions associated with imagining a successful outcome.
Decreasing external (social) pressure	Learning to say “no” in an assertive manner	Participants are briefly taught on what constitutes assertive behavior, including sub‐assertive behavior. Next, they receive recommendations on how to respond to requests they do not want to comply with, including keeping one's response brief, optionally providing further explanation, and using the “broken record technique,” which involves calmly and persistently repeating your position.
	Formulating “I” statements	An “I” statement is a communication technique used to express one's thoughts or feelings in a nonconfrontational manner. It typically starts with “I feel,” “I think,” or “I believe,” followed by a description of the emotion or thought, and concludes with a specific request or suggestion. For example, instead of saying, “You always walk too fast for me,” which can come across as accusatory and may lead to defensiveness, one could say, “I feel stressed because I cannot keep up with your pace. Could you slow down a bit?”
Having a backup plan in case of gait difficulties	Carefully planning out the walk beforehand using problem‐solving	This strategy is based on the problem‐solving training protocol by O'Donohue and Fisher (2008). The therapist and participants together prepare for a challenging route, devising a strategy to tackle any obstacles along the way. Any bottlenecks that are present on the route are first identified. Next, the therapist and participants brainstorm about every possible solution that comes to mind to overcome these obstacles, also those that seem not to be the best. Participants are encouraged to think broadly and list the pros and cons of any of these solutions. Once a list of possible solutions is generated, the therapist and participants evaluate which solution is best for overcoming the obstacles, which is then tried out.
	Other categories of compensation strategies	Participants may also choose a strategy from one of the other six categories of compensation strategies that were identified by Nonnekes et al. (2019 ). These strategies may provide participants with a backup plan in case of gait difficulties, thereby reducing participants' fear of FOG by giving them a sense of control over the situation. Examples of strategies include the use of external or internal cues while walking (e.g., walking on the pace of a metronome or counting in your head), adopting a different walking pattern (e.g., lifting the knees higher), or using a walking aid. See Nonnekes et al. (2019 ) for a further description of these strategies.

#### 2.7.3 Sessions 3 and 4: Subjective Evaluation of Strategy Efficacy

During the last two sessions, the subjective effects of the strategies will be evaluated. For this purpose, the diary that the participants were asked to fill in will be used. The therapist begins by asking the participants to give a general comment on how the selected strategies impacted their anxiety, stress, and unhelpful thoughts related to FOG, as well as the impact this had on FOG. Next, participants are asked to provide specific examples of moments when they practiced the strategies in situations relevant to them. If participants encountered difficulties while practicing the strategies, the therapist and participants explore ways to resolve these. For instance, in the case of difficulty practicing the strategies regularly, it can be considered whether the partner or caregiver may provide additional support in this regard. The strategies that were effective will be retained, and those that were unfeasible or not sufficiently effective will be replaced by alternative strategies.

In the final session, it is also discussed whether the participants want to keep using the strategies after the intervention has ended. If this is the case, the therapist and participants engage in a discussion that includes questions such as which strategies they wish to continue using and how they plan to integrate these strategies into their everyday lives. If they have a partner or caregiver, it is also discussed how they can support the participants in maintaining the use of the strategies. Lastly, the utility of the intervention is discussed, including what parts the participants found particularly helpful and which parts they perceived to be less useful.

### 2.8 Procedure and Assessments

All measurements will be performed in the home setting of the participants. The intervention group will be assessed three times: at baseline (T0), post‐intervention (T1), and after a 10‐week follow‐up period (T2). The waitlist control group is assessed four times: at baseline (T0), after the waiting period (T1), post‐intervention (T2), and after a 10‐week follow‐up period (T3), see Figure 1. The assessments will be conducted according to a standardized protocol and will be performed by a trained assessor. All measurements will be performed at the same time of the day, in a subjective regular dopaminergic ON‐state, always after an identical interval after intake of the participant's own dopaminergic medication. The use of walking aids is allowed during the gait tasks, provided that the same aid is used consistently across all assessment time points.

To promote adherence to the study protocol, participants will receive a reminder via email before every appointment. The day on which the appointments are scheduled will be discussed with the participants. If an appointment has to be canceled for any reason, a new appointment is scheduled as closely as possible to the original date. Additionally, any adjustments to medication and DBS settings will be logged throughout the study and reported descriptively. Changes occurring during the intervention will not be included as covariates in the analyses. Given the relatively short duration of the intervention, substantial changes in DBS settings or medication dosage are considered unlikely; therefore, the risk of confounding is expected to be minimal. Participants are allowed to continue with usual care.

### 2.9 Demographic and Clinical Assessments

Demographics and clinical characteristics will be collected at baseline to characterize the cohort and to explore their potential as predictors of treatment response (i.e., change in percentage of time spent freezing during the neutral condition of the walking course). Demographic variables include age, sex, presence of a caregiver, disease duration (i.e., number of years since diagnosis), and medication status, including levodopa equivalent daily dose.

Clinical assessments consist of Part III of the Movement Disorder Society‐sponsored Unified Parkinson's Disease Rating Scale (MDS‐UPDRS) to assess disease severity (Goetz et al. 2008). Cognitive function is assessed using the Montreal Cognitive Assessment (MoCA) (Nasreddine et al. 2019). The Beck Depression Inventory is administered to evaluate the presence of depression (Richter et al. 1998). The level of anxiety is assessed with the Parkinson Anxiety Scale (Leentjens et al. 2014). Last, the Coping Inventory for Stressful Situations‐21 (CISS‐21) is administered to measure coping styles that individuals use when dealing with stressful situations (Cohan et al. 2006).

### 2.10 Primary Outcome

The primary outcome is the percentage of time spent frozen during a walking course that includes anxiety‐ and stress‐related FOG hotspots in the participants' home setting. Prior to the walking tasks, participants will be asked to identify locations or situations in their home environment where they frequently experience FOG and where they perceive anxiety or stress to play a role. Specifically, participants will first be asked to indicate locations and situations in which they regularly experience FOG in their home environment. For each situation, they will then be asked whether they feel that anxiety or stress contributes to these episodes by asking: “Do you notice that anxiety or stress contributes to experiencing freezing of gait in this place/situation?” The course is performed three times under comfortable walking speed and is created in a way that each trial will last at least 1 min. During walking, the assessor walks alongside participants to provide manual assistance should they become unstable; in such cases, the trial is repeated.

Participants will be videotaped while walking the course by the assessor using a handheld camera. The assessor will walk consistently beside the participant, approximately 1 m away, holding the camera at chest height to keep the participant's whole body visible up to the shoulders, with a clear view of the lower limbs and feet. After the assessments, the videos will be annotated by the assessor using the FOG annotation tool ELAN to calculate the percentage of time spent frozen during the task, based on the new consensus‐based “technical” definition for FOG (Gilat et al. 2026; Brugman et al. 2004). The start of each trial is verbally indicated by the assessor “Start walking”; the end of each trial is defined by the moment the participants sit down in a chair.

### 2.11 Secondary Outcomes

The secondary outcomes consist of performing the walking course (see primary outcome) under elevated stress conditions, a standardized FOG‐provoking protocol, and questionnaires. In addition, heart rate is monitored during the personalized walking course and during rest. These secondary outcomes will be assessed at all time points. The following secondary outcomes will be included:

#### 2.11.1 Walking Course With Stress Manipulation

The walking course will be performed under elevated stress conditions by adding time pressure and an element of social evaluation, based on the Trier Social Stress Test, in which participants are given a very short period of time to prepare for a presentation in front of a committee. This test is considered the gold standard for eliciting stress in controlled settings (Allen et al. 2017). The walking course with stress manipulation will be performed three times, following the exact same route as the baseline course. Again, the percentage of time frozen during the task is used as the outcome variable.

After completing the baseline walking course, participants are told they have to complete the same walking course, but they have to improve their performance. To induce time pressure, they are instructed to complete the walking course 20% faster than during the baseline condition, which they are told other participants can achieve. Before starting the task, they are then told that the video recordings will be sent to three experienced researchers who will critically evaluate their performance and compare it to that of other participants. The hotspots and intended walking route will be kept as similar as possible between lower and higher stress conditions and will again be performed three times. The instruction to participants will be: “Now you need to walk the route three times again, but this time you have to perform better. The goal is for you to walk the route 20% faster than before. Other participants have managed to do this, so you should be able to as well. Additionally, you will be filmed again. This time, the footage will be sent to three experienced researchers who will critically assess your performance. The researchers will evaluate your effort and how well you performed compared to the other participants.”

#### 2.11.2 Standardized FOG‐Provoking Protocol

In addition to the personalized walking course, participants complete a standardized FOG‐provoking gait protocol, which is administered once during the home‐based assessments. This protocol consists of eight tasks in which participants have to perform actions that frequently elicit FOG (Ehgoetz Martens KH et al. 2025). These include quickly walking a few meters back and forth (while carrying an object), making rapid 360° turns, shuffling around a 50 × 50 cm^2^ on the ground, and passing a doorway. Some of the tasks are performed a second time during which the participants are asked to perform a cognitive (serial subtraction) task simultaneously.

#### 2.11.3 Heart Rate

To assess participants' physiological arousal levels before and during the walking course, heart rate will be derived from ambulatory electrocardiogram (ECG) recordings. Heart rate will be used to examine whether the intervention reduces physiological arousal during the walking course, compared to the waitlist group, and whether this effect is maintained at follow‐up. Resting heart rate will be measured once prior to the neutral condition and once prior to the manipulated stress condition, each time over a 1‐min period while participants are seated comfortably. Heart rate during walking is recorded for the total duration of each trial. Mean heart rate during walking will be calculated for each trial in both conditions of the walking course. These values will then be averaged across trials within each condition to obtain one mean heart rate per condition. For this purpose, 3‐lead ECG signals are recorded for heart rate monitoring prior to and during the walking course, using a portable system (Mobi8‐4b4as, Twente Medical Systems International, Oldenzaal, The Netherlands), with a sampling rate of 1024 Hz. Data analysis of the ECG signals will be performed with MATLAB (The MathWorks Inc., Natick, MA). A 2–20‐Hz passband filter will be applied to the raw ECG signal to minimize low‐frequency drift and high‐frequency noise. Subsequently, R‐peaks will be identified automatically based on their amplitude and temporal spacing. Peaks that have a minimal amplitude of 0.9 times the standard deviation of the ECG signal will be automatically detected. A minimum peak distance of 0.4 s is applied to enhance peak detection accuracy. Visual inspection of the automatically detected peaks is then performed to ensure peak detection was successful. If short‐lasting artifacts prevent an accurate calculation of the minimum peak height from the whole trial, a clean, shorter segment is manually selected to calculate the minimal peak height. In the cases where short‐lasting artifacts mask the R‐peaks, no R‐peak will be registered. The R‐R intervals will be used to calculate heart rate as 60 divided by the mean normal‐to‐normal R‐R interval.

#### 2.11.4 Subjective Anxiety or Stress During the Walking Course

After completing the three trials of each walking condition, participants will rate the level of anxiety or stress they experienced during walking using a 10‐point numeric scale.

#### 2.11.5 Questionnaires

In addition, several questionnaires are administered during each visit. Participants will complete the questionnaires themselves. However, the assessor assists in completing the questionnaires if participants are unable to do so themselves. The following questionnaires will be administered:

The *Parkinson Anxiety Scale* is a 12‐item questionnaire that has been specifically developed and validated for measuring anxiety symptoms in people with PD (Leentjens et al. 2014). It contains three subscales that measure persistent anxiety, episodic anxiety, and avoidance behavior.

The *Perceived Stress Scale* is a self‐reported questionnaire that consists of 10 items that measure the degree to which participants perceived stress in the last month (Cohen et al. 1983). Participants indicate how often they were bothered by thoughts and feelings that are related to stress.

The *Gait‐Specific Attention Profile* provides a measure of multiple psychological factors that are associated with anxiety and implicated in influencing gait performance (Young et al. 2020). The scale contains 11 items that pertain to the following psychological factors: conscious movement processing, fall‐related ruminations, processing inefficiencies, and anxiety.

The *Updated Perceived‐Control Over Falling* is a 4‐item scale that asks participants about their perceptions regarding their ability to manage their risk of falling (Ellmers et al. 2023). Participants indicate to what extent they agree or disagree with each statement on a 5‐point Likert scale.

The *Rosenberg Self‐esteem Scale* is a 10‐item scale to assess global self‐esteem by measuring both positive and negative feelings about oneself (Robins et al. 2001). Participants indicate whether they agree or disagree with each statement using a 4‐point Likert scale.

The *Parkinson's Disease Questionnaire* (*PDQ‐39*) is a disease‐specific instrument that is developed to measure relevant aspects that pertain to Quality of Life in people with PD (Jenkinson et al. 1997). It contains 39 items that cover eight domains of life, such as mobility, social support, and activities of daily living.

The *New Freezing of Gait Questionnaire* is a self‐reported measure that contains nine questions about FOG. These involve the presence and severity of FOG, as well as the impact it has on daily life (Nieuwboer et al. 2009).

Participants are asked to indicate the degree to which anxiety or stress has an impact on the occurrence of FOG, using a Numeric Rating Scale (NRS) ranging from 0 to 10.

### 2.12 Sample Size

A sample size calculation was performed in GPower to determine the sample size required to reach a statistical power of 0.95 at an alpha corresponding to 0.01 (Faul et al. 2007). The power calculation was based on a two‐tailed independent *t*‐test. Based on previous intervention studies and pilot data that used the percentage of time frozen as primary outcome measure (Walton et al. 2018; Barthel et al. 2018), a mean reduction of the time spent frozen of 27.5% (SD: 15%) is expected in the intervention group, and 5% (SD: 15%) in the control group (due to the Hawthorne effect), corresponding to an effect size of 1.5. To reach the desired statistical power of 0.95 with the expected effect size, each group should contain 18 participants. To compensate for a potential dropout of 10%, 20 participants will be included per group, resulting in a total sample size of 40 participants.

### 2.13 Statistical Analysis

Statistical analysis will be performed in IBM SPSS 29 (SPSS Inc., Chicago, IL). The effect of the intervention on the primary and secondary outcome variables directly post‐intervention will be tested using ANCOVA. Post‐intervention measurements will be used as dependent variables with pre‐intervention (baseline) measurements as the covariate to control for baseline differences between the groups. This approach provides an adjusted estimate of the intervention effect, accounting for baseline group differences and thereby improving statistical power. Group (intervention vs. waitlist) is used as a between‐subjects factor. The effect of time will be tested separately for the primary and each secondary outcome by merging both groups and performing repeated measures ANOVAs with time as the within‐subject factor (intervention group: T0, T1, and T2; control group: T1, T2, and T3). Subsequently, post hoc paired *t*‐tests or Wilcoxon tests will be performed in case of a significant time effect to identify which time points differ from each other. First, data will be checked for normality using Shapiro–Wilk. Then, a series of independent samples *t*‐tests (or Mann–Whitney tests when appropriate) will be used to assess differences between the groups. Finally, to evaluate key determinants of the intervention, a stepwise linear regression will be performed to assess the relationship between participant characteristics and the change in the percentage of time frozen following the intervention.

## 3 Discussion

The aim of the current study is to evaluate the effectiveness of a tailored nonpharmacological intervention to reduce anxiety‐ and stress‐related FOG. While cognitive and affective deficits can modulate FOG (Martens et al. 2014; Heremans et al. 2013), only a few studies have explored the potential of targeting these factors to reduce FOG. One randomized controlled trial examined the efficacy of cognitive training to reduce FOG and showed that the intervention resulted in a significant reduction in FOG severity in the ON‐state of participants (Walton et al. 2018). To date, there have been few studies that have explored the potential of reducing anxiety and stress to reduce FOG. While one study effectively established that an 8‐week yoga intervention significantly improved participants' self‐reported severity of FOG (Van Puymbroeck et al. 2018), it is unclear whether these improvements stemmed from the meditation aspect or the physical rehabilitation aspect of the intervention. In addition, the study did not examine the effect that the intervention had on anxiety and stress levels. Instead, the current study examines whether a personalized intervention that directly targets anxiety and stress might reduce FOG.

An important novel aspect of the current study is the personalized treatment approach that is used to reduce anxiety‐ and stress‐related FOG. The importance of using a tailored approach to gait rehabilitation has been shown in previous work in which the use and patient‐rated efficacy of compensation strategies was evaluated systematically (Tosserams et al. 2020, 2022). The efficacy of compensation strategies varies significantly from person to person, with some individuals experiencing positive effects while others may see no benefit or even worsening of their gait. Moreover, the effectiveness of compensation strategies differs between situations in which they are applied. For example, when experiencing social anxiety related to freezing, a different approach is likely needed compared to a situation where someone experiences FOG that is related to time pressure. In this study, personalization is achieved by examining the specific situations and environments in which participants experience anxiety‐ or stress‐related FOG. Subsequently, participants are offered tailored behavioral strategies that they can apply in these contexts to reduce anxiety‐ and stress‐related FOG.

This study is one of the first studies that adopts a home‐based approach to measure FOG. We will objectify the occurrence of FOG using FOG‐provoking hotspots in the home environment of the participants. Since the intervention will be tailored based on the person and context in which FOG occurs, this setup allows for investigating the effectiveness of the strategies in the situations for which they were selected. A previous study that applied a similar home‐based approach to measure FOG successfully established that a cueing device was able to reduce FOG severity, which highlights the potential of measuring FOG in a home environment (Zoetewei et al. 2024). Additionally, adopting a home‐based approach may enable us to measure FOG more effectively, as FOG is difficult to provoke in controlled settings such as the doctor's office or during laboratory‐based gait assessments (Mancini et al. 2019).

We will include both physiological and subjective measures of arousal to assess whether changes in anxiety are associated with changes in FOG. To obtain an objective measure of physiological arousal during the walking course, ambulatory ECG recordings will be used to derive heart rate data, providing real‐time and objective information on arousal prior to and during the walking course. To obtain a subjective measure of anxiety, participants will be asked to rate their level of anxiety immediately after completing the walking course and to indicate the extent to which anxiety influenced the occurrence of FOG over the past week.

In addition to investigating the effectiveness of the intervention, this study also examines what the key factors are that contribute to the response to the intervention. We anticipate that several factors will influence the effectiveness of the intervention. First, the severity of PD may affect the extent to which the intervention leads to a reduction in the percentage of FOG, since several symptoms that may worsen as the disease progresses might hinder the extent to which the participants can engage with the intervention material, including apathy, mental fatigue, and cognitive dysfunction (Kurlawala et al. 2021). Cognitive functioning may affect how well participants can participate in the sessions and follow the treatment plan, so we expect this to be an independent predictor of the outcome of the intervention (Metts et al. 2018). Other determinants include depression because this is often accompanied by a general lack of motivation, concentration difficulties, and fatigue (American Psychiatric Association D and Association AP 2013), and utilization of a passive coping style since it hampers active engagement during the treatment (Lazarus and Folkman 1984). Finally, we expect that participants with a partner or caregiver may benefit more from the intervention, as this likely promotes adherence to the treatment protocol (Martire et al. 2004).

Several challenges of the present trial design should be acknowledged. First, although participants are encouraged to maintain stable dopaminergic medication and DBS settings, clinically indicated changes are allowed during the trial, which may influence FOG outcomes independent of the intervention. However, such changes are unlikely to systematically differ between groups, given the randomized design. In addition, adherence to the practiced strategies outside the intervention sessions will not be formally quantified, which may contribute to variability in treatment response. Furthermore, due to the nature of the intervention, neither participants nor assessors can be blinded, meaning that expectancy or observer‐related biases cannot be fully ruled out. The experimentally induced stress condition represents another potential limitation: although based on established stress paradigms and applied successfully in our pilot, this task has not yet been formally validated in people with PD, nor has its ability to provoke FOG been systematically evaluated in this population. Lastly, the sample size calculation was informed by effect estimates derived from previous cueing interventions and our pilot data, in the absence of prior randomized trials specifically targeting anxiety‐related FOG with a behavioral intervention, and may therefore be unable to detect smaller but clinically relevant effects.

The TACKLING‐FOG study aims to contribute to evidence‐based and personalized rehabilitation of gait impairments in people with PD. The study may highlight the importance of incorporating anxiety and stress management into the treatment approach for FOG. If the Managing the Mental State intervention significantly reduces the impact of anxiety and stress on FOG, it can be added to the repertoire of potential treatments for FOG.

## 4 Trial Status

Participant recruitment has started in March 2024 and is currently ongoing. The first measurements have started in the first week of April 2024. This study protocol is based on protocol version 6, dated March 29, 2024.

## Author Contributions

J.N., W.R.Y., and A.T. contributed to the conception and design of the study. The acquisition of funding from The Gossweiler Foundation was led by J.N., W.R.Y, and A.T. The protocol was finalized with the contributions of A.A.D., R.C.H., B.R.B., and A.H. Participant inclusion, data collection and study management are performed by G.V. J.N. is the principal investigator. All authors contributed to the writing and/or editing of the manuscript and approved the final version.

## Funding

The study is funded by the Jacques und Gloria Gossweiler Foundation. The funders are not involved in the development of the protocol, the execution, analysis, or interpretation of the study data.

## Ethics Statement

The study has received ethical approval from the accredited medical ethical committee METC Oost‐Nederland, the Netherlands (NL85217.091.23). Any protocol modifications will be notified to the committee via an amendment. Also, the trial has been registered on Clinicaltrials.gov (NCT06302309). All participants will sign informed consent prior to data collection and obtained by the assessor (MH) on the day of the first measurements.

## Conflicts of Interest

The authors declare no conflicts of interest.

## Data Availability

The data of this study will be available from the corresponding author upon reasonable request once the findings of the study are published.
